# Racial variations in tooth pain and care-seeking in adolescents in Malaysia

**DOI:** 10.1038/s41405-021-00058-5

**Published:** 2021-01-19

**Authors:** Kangjie Tay, Cassandra Li Jean Beh, Muneer Gohar Babar, Ting Jing Kweh, Ekta Priya, Allan Pau

**Affiliations:** School of Dentistry, International Medical University, Kuala Lumpur, Malaysia

**Keywords:** Dental epidemiology, Oral-health-related quality of life

## Abstract

**Objective:**

Tooth pain among adolescents is a common event that impacts substantially on quality of life. The purpose of this paper is to explore the role of race in the tooth pain experience and associated care-seeking.

**Design:**

A cross-sectional questionnaire survey was conducted on 14–18 years from four different public schools in Kuala Lumpur. Socio-demographic, pain symptoms, and social impacts data were collected as well as medication use and visiting a dentist for tooth pain.

**Results:**

Of 1473 questionnaires distributed, 1452 (98.6%) completed questionnaires were returned, with 269 (18.5%) reported having experienced tooth pain in the past 1 month. Those who identified as Indian (26.1%) were more likely to report tooth pain compared to Chinese (16.5%) and Malay (20.9%). In regression analysis, no factors were identified as independently associated with medication use, but Chinese and Indians compared to Malays, and those expressing difficulty sleeping were more likely to report visiting a dentist for treatment.

**Conclusion:**

Racial variations in the tooth pain experience and care-seeking have been identified. This may be related to socio-economic status, but further research is needed on the role of culture-related care-seeking and accessibility of dental services.

## Introduction

Tooth pain due to permanent caries has been reported to exceed 200 million incident cases worldwide in 2013. Also referred to as toothache, dental pain or odontogenic pain, it is defined as pain initiating from the teeth or their supporting structures, the mucosa, gingiva, maxilla, mandible or periodontal membrane.^1^ Prevalence estimates of tooth pain have been reported to affect a range of children and adolescents worldwide, partly depending on the time intervals in which it is experienced and the age group of the samples. For example, tooth pain in the past year has been reported to affect 54.2% of 12–15-year-olds in China,^2^ whereas tooth pain in the past 6 months affected 21.8% of 11–17 year-olds in Brazil,^3^ tooth pain in the past 3 months affected 36.4% of 10–19 year-olds in Tanzania,^4^ and tooth pain in the past month affected 30.4% of 11–14-year-olds in Pakistan.^5^

Substantial proportions of children and adolescents with tooth pain report impact on social functioning,^6,7^ with difficulty in eating and sleeping being the more commonly reported.^8^ The use of medication for tooth pain relief is common.^9,10^ Indeed tooth pain has been reported to account for most hospital admissions for unintentional paracetamol overdose.^11,12^ The use of dental services for tooth pain has been reported to be associated with experience of symptoms and impacts.^5,13^ Tooth pain has also been reported to account for most of emergency dental visits in the hospital setting.^14,15^

That tooth pain in children and adolescents is common and its social impact substantial is widely acknowledged. Socio-economic, family and individual factors associated with tooth pain have also been widely reported. Increasingly, racial disparity in the tooth pain experience has also been documented. For example, Brazilian Indigenous children are more likely than White to report tooth pain,^16^ New Zealand Maori more likely than European,^17^ Australian Indigenous more likely than White.^18^ Freire et al. (2018) reported that Brazilian Black and Indigenous 5-year-olds are more likely to report oral health related social impact when compared to White children, but this was not significant after controlling for pain experience, caries status, and household income.^7^ Care-seeking patterns in relation to tooth pain and its association with race, however, is less well established, although disparities in access to dental care is evident when comparing American Black, Hispanic, and Native American children to White children,^19^ and similarly in Brazilian 13–17 year-olds.^20^ The research literature on race and tooth pain is growing, and more research on race, ethnicity and socio-economic status, and how these complex constructs affect healthcare and health outcomes in children and adolescents has been called for.^21^ The conceptualisation and definition of race and ethnicity are subjects of debate in the health research literature,^22^ however, a useful reference point is given by Bhopal.^23^ Race is generally defined based on physical features reflecting ancestry and genetics but has increasingly incorporated a common social and political heritage. It has increasingly been displaced by ethnicity, which is perceived to be based on culture, language, diet, religion, ancestry, and physical features. In this conceptualisation, ethnicity subsumes race, but in certain contexts, such as in Malaysia, the use of the term race is preferred and commonly used colloquially and in official documentation.^24^ In Malaysia, racial categorisation has been reported to be based on physical characteristics, such as skin colour, with roots in historical and political identification of social groups. In the present-day social context, racial groups with darker skin are perceived to face discrimination, but irrespective of skin colour, racial identity is used as a basis for bestowing certain political and socio-economic opportunities. While the concept of ethnicity has developed with the incorporation of cultural and socio-economic dimensions into race, the concept of race can have other connotations and is commonly applied in some parts of the world.

The role of race in health outcomes and care-seeking behaviours is therefore an important area of study. In this paper, we investigate the role of race in the tooth pain experience, as well medication use and dental care-seeking for the tooth pain experience. We analyse the role of pain intensity and anatomical functioning, and impact on social functioning on medication use and dental care-seeking, in order to determine whether race is an independent predictor of medication use and dental care-seeking in Malaysian adolescents experiencing tooth pain.

## Methodology

A cross-sectional questionnaire survey was conducted in 2014 among 14–18-year-old adolescents attending four public (government) schools in a district in the outskirt of Kuala Lumpur, Malaysia.

The survey was independently reviewed and approved by the institutional Joint Committee for Ethics and Research. Permission to conduct the survey was sought from the principals of the four schools. The investigators arranged a face-to-face meeting with each principal to discuss the purpose, scope and importance of the study and to address any queries. Assurance that the students would be able to comprehend the questionnaire items in English was established. A mutually convenient date was identified with each school to conduct the survey.

The survey included students aged between 14 and 18 years, had no childhood disease that may possibly affect their oral health and were willing to complete the questionnaire. Students who did not meet the set inclusion criteria were excluded. The investigators provided oral and written explanation of the purpose and conduct of the survey and reassured the students that their participation was voluntary, and information gathered would remain confidential. Written information and consent forms were given to the students to take home for their parents to scrutinise and provide consent.

The students were introduced to the Modified-Dental Pain Questionnaire (DePaQ)^5^ and the Child Oral Impacts on Daily Performances (Child-OIDP).^25^ The Modified DePaQ consists of 16 items on dental pain characteristics such as intensity, duration and associated symptoms, and the Child-OiDP focusses on impact on playing, relaxing or going to school in the past month. Data on school attended, age, sex and race were also collected, as well as medication use and dental visits for self-reported tooth pain. The questionnaires were piloted in one of the schools to ensure the feasibility and acceptability of using them in this population. The questionnaires were distributed to the students during a class with permission from the teacher. The investigators were present to address any queries. Completion of the questionnaires took around 20 min. An oral health education activity on oral hygiene, toothbrushing and diet control was delivered.

Statistical analyses were performed using Statistical Package for Social Sciences (SPSS for Windows, version 21.0, SPSS Inc. Chicago, IL, USA) program. Distributions of tooth pain were calculated for school, age, sex, and race categories. Chi-square analysis was carried out to test for association. Of those reporting tooth pain, association of medication use and dental visits for tooth pain with pain symptoms/anatomical functioning and social impacts, and demographic characteristics was analysed using the Chi-square test. The level of statistical significance was set at *p* = 0.05 for all tests. Multivariate hierarchical analyses from distal to proximal determinants on three levels were carried out: socio-demographic characteristics; pain characteristics/anatomical functioning; and impact on social functioning. At each level, the backward stepwise method was used for the selection of variables with a *p* value < 0.20 in the bivariate analysis.

## Results

Of 1473 questionnaires distributed, 1452 (98.6%) completed questionnaires were returned, with 269 (18.5%) reporting tooth pain in the past 1 month. School of attendance, sex and age were not associated with tooth pain, but 29 (26.1%, 17.8–34.4) subjects who identified themselves as Indian were more likely to report tooth pain experience when compared to 131 (16.5%, 13.9–19.1) who identified themselves as Chinese (Table 1).

**Table 1:** Number (%) of students who reported pain in tooth in the past 1 month (*N* = 1452).

	No	Yes	Total	*p* value
*School*				
School A	371 (84.1)	70 (15.9)	441 (100.0)	0.211
School B	360 (81.1)	84 (18.9)	444 (100.0)	
School C	307 (81.0)	72 (19.0)	379 (100.0)	
School D	145 (77.1)	43 (22.9)	188 (100.0)	
*Sex*				
Boy	581 (81.6)	131 (18.4)	712 (100.0)	0.903
Girl	602 (81.4)	138 (18.6)	740 (100.0)	
*Age in years*				
14	341 (80.6)	82 (19.4)	423 (100.0)	0.452
15	433 (83.8)	84 (16.2)	517 (100.0)	
16	167 (81.1)	39 (18.9)	206 (100.0)	
17	183 (78.2)	51 (21.8)	234 (100.0)	
18	59 (81.9)	13 (18.1)	72 (100.0)	
*Race*				
Malay	405 (79.1)	107 (20.9)	512 (100.0)	0.007
Chinese	663 (83.5)	131 (16.5)	794 (100.0)	
Indian	82 (73.9)	29 (26.1)	111 (100.0)	
Others	33 (94.3)	2 (5.7)	35 (100.0)	
Total	1183 (81.5)	269 (18.5)	1452 (100.0)	

Of 269 who reported tooth pain, 249 (92.6%) completed questionnaires were analysed for demographic characteristics, pain characteristics, and social impacts associated with pain medication use and dental visits for tooth pain in the past 1 month. Fifty-one (20.5%, 15.7–26.0) students reported using pain medication and 65 (26.1%, 20.8–32.0) visiting the dentist. Medication use and dental visits were not statistically significantly associated with school, sex, and age. Indians (10, 37.0%, 19.4–57.6) were more likely to report medication use compared to Chinese (17, 14.0%, 8.4–21.5), whereas Indians (10, 37.0%, 19.4–57.7) and Chinese (38, 31.4%, 23.3–40.5) were more likely to visit a dentist compared to Malays (17, 16.8%, 10.1–25.6). Of those who reported medication use, 26 (52.0%, 36.6–65.3) subjects reported dental visits compared to 39 (19.7%, 14.4–25.9) who reported no medication use (Table 2).

**Table 2:** Numbers (%) of students who had used medication and visited a dentist for tooth pain in the past 1 month, distributed by school, sex, age and race (*N* = 249).

	Medication use				Dental visits				Total
	No	Yes	CI % (LL,UL)	*p* value	No	Yes	CI (LL,UL)	*p* value	
*School*									
School A	52 (85.2)	9 (14.8)	7.0, 26.1	0.276	48 (78.7)	13 (21.3)	11.9, 33.7	0.184	61
School B	64 (79.0)	17 (21.0)	12.7, 31.4		60 (74.1)	21 (25.9)	16.8, 36.9		81
School C	52 (81.3)	12 (18.8)	10.0, 30.4		50 (78.1)	14 (21.9)	12.5, 34.0		64
School D	30 (69.8)	13 (30.2)	17.2, 46.1		26 (60.5)	17 (39.5)	25.0, 55.6		43
*Sex*									
Boy	90 (76.3)	28 (23.7)	16.4, 32.4	0.228	88 (74.6)	30 (25.4)	17.9, 34.3	0.816	118
Girl	108 (82.4)	23 (17.6)	11.5, 25.1		96 (73.3)	35 (26.7)	19.4, 35.1		131
*Age in years*									
14	60 (82.2)	13 (17.8)	9.8, 28.5	0.736	50 (68.5)	23 (31.5)	21.1, 43.4	0.687	73
15	63 (78.8)	17 (21.3)	12.9, 31.8		63 (78.8)	17 (21.3)	12.9, 31.8		80
16	25 (71.4)	10 (28.6)	14.6, 46.3		25 (71.4)	10 (28.6)	14.6, 46.3		35
17	40 (81.6)	9 (18.4)	8.8, 32.0		37 (75.5)	12 (24.5)	13.3, 38.9		49
18	10 (83.3)	2 (16.7)	2.0, 48.4		9 (75.0)	3 (25.0)	5.5, 57.2		12
*Race*									
Malay	77 (76.2)	24 (23.8)	15.9, 33.3	0.016	84 (83.2)	17 (16.8)	10.1, 25.6	0.019	101
Chinese	104 (86.0)	17 (14.0)	8.4, 21.5		83 (68.6)	38 (31.4)	23.3, 40.5		121
Indian	17 (63.0)	10 (37.0)	19.4, 57.6		17 (63.0)	10 (37.0)	19.4, 57.7		27
Total	198 (79.5)	51 (20.5)			184 (73.9)	65 (26.1)			249

Confidence interval for proportion of students who used medication- lower limit-15.7, upper limit- 26.0.

Confidence interval for proportion of students who visited dentist- lower limit-20.8, upper limit- 32.0.

Of the pain characteristics/anatomical functioning impacts, pain intensity and difficulty on chewing/eating were statistically significantly associated with pain medication use, whereas pain intensity, swollen gums and difficulty swallowing were statistically significantly associated with dental visits (Table 3).

**Table 3:** Number (%) of students who had taken medicine and visited a dentist for tooth pain in the past 1 month, distributed by pain symptoms.

	Medication use				Dental visits				Total
	No	Yes	CI (LL,UL)	*p* value	No	Yes	CI (LL,UL)	*p* value	
*Pain duration*									
Less than 1 week	140 (79.5)	36 (20.5)	14.8, 27.2	0.707	134 (76.1)	42 (23.9)	21.6, 36.8	0.269	176 (70.7)
1 week or longer, but less than 1 month	34 (82.9)	7 (17.1)	7.2, 32.0		30 (73.2)	11 (26.8)	14.2, 42.9		41 (16.5)
1 month or longer	24 (75.0)	8 (25.0)	11.5, 43.4		20 (62.5)	12 (37.5)	21.1, 56.3		32 (12.8)
*Pain intensity*									
Slight	81 (82.7)	17 (17.3)	10.4, 26.3	0.002	79 (80.6)	19 (19.4)	12.1, 28.6	0.001	98 (39.4)
Moderate	106 (82.2)	23 (17.8)	11.7, 25.6		97 (75.2)	32 (24.8)	17.6, 33.1		129 (51.8)
Severe	11 (50.0)	11 (50.0)	28.2, 71.8		8 (36.4)	14 (63.6)	40.7, 82.8		22 (8.8)
*Pain is constant*									
No	163 (81.1)	38 (18.8)	13.7, 25.0	0.207	153 (76.1)	48 (23.9)	18.1, 30.4	0.102	201 (80.7)
Yes	35 (72.9)	13 (27.1)	15.3, 41.9		31 (64.6)	17 (35.4)	22.2, 50.5		48 (19.3)
*Pain localised*									
In one tooth or one spot	176 (80.0)	44 (20.0)	14.9, 25.9	0.740	163 (74.1)	57 (25.9)	20.3, 32.2	0.940	220 (88.4)
In more than one tooth or over a big area	22 (75.0)	7 (24.1)	10.3, 43.5		21 (72.4)	8 (27.6)	12.7, 47.2		29 (11.6)
*Pain worse on chewing/eating*									
No	138 (83.6)	27 (16.4)	11.0, 22.9	0.024	128 (77.6)	37 (22.4)	16.3, 29.6	0.064	165 (66.3)
Yes	60 (71.4)	24 (28.6)	19.2, 39.5		56 (66.7)	28 (33.3)	23.4, 44.5		84 (33.7)
*Pain worse with cold drink/food*									
No	126 (81.3)	29 (18.7)	12.9, 25.8	0.374	120 (77.4)	35 (22.6)	16.3, 30.0	0.104	155 (62.2)
Yes	72 (76.6)	22 (23.4)	17.1, 35.6		64 (68.1)	30 (31.9)	22.7, 42.3		94 (37.8)
*Gums swollen*									
No	164 (81.2)	38 (18.8)	13.7, 24.9	0.176	156 (77.2)	46 (22.8)	17.1, 29.2	0.013	202 (81.1)
Yes	34 (72.3)	13 (27.7)	15.6, 42.6		28 (59.6)	19 (40.4)	26.4, 55.7		47 (18.9)
*Painful tooth felt loose*									
No	139 (79.8)	35 (20.1)	14.4, 26.9	0.825	133 (76.4)	41 (23.6)	43.9, 61.0	0.164	174
Yes	59 (78.7)	16 (21.3)	12.7, 32.3		51 (68.0)	24 (32.0)	21.7, 43.8		75
*Difficulty swallowing*									
No	169 (81.6)	38 (18.4)	13.3, 24.3	0.065	160 (77.3)	47 (22.7)	17.21, 29.0	0.007	207
Yes	29 (69.0)	13 (31.0)	17.6, 47.0		24 (57.1)	18 (42.9)	27.7, 59.0		42
*Painful tooth felt like it was sticking out*									
No	167 (81.1)	39 (18.9)	13.8, 25.0	0.185	155 (75.2)	51 (24.8)	19.0, 31.2	0.289	206
Yes	31 (72.1)	12 (27.9)	15.3, 43.7		29 (67.4)	14 (32.6)	19.1, 48.5		43
*Difficulty sleeping*									
No	160 (84.2)	30 (15.8)	10.9, 21.8	0.001	157 (82.6)	33 (17.4)	12.3, 23.5	0.001	190
Yes	38 (64.4)	21 (35.6)	23.6, 49.1		27 (45.8)	32 (54.2)	40.8, 67.3		59
Total	198 (79.5)	51 (20.5)	15.7, 26.0		184 (73.9)	65 (26.1)	20.8, 32.0		249

Of the social functioning impacts analysed, difficulty relaxing/sleeping and cleaning mouth were statistically significantly associated with pain medication use, whereas difficulty playing, eating/drinking, relaxing/sleeping, speaking/pronouncing, cleaning mouth, and smiling/laughing were statistically significantly associated with dental visits (Table 4).

**Table 4:** Number (%) of students who had used medicine and visited a dentist for tooth pain in the past 1 month, distributed by social impacts.

	Medication use				Dental visits				Total
	No	Yes	CI (LL,UL)	*p* value	No	Yes	CI (LL,UL)	*p* value	
*Difficulty playing*									
No	163 (81.5)	37 (18.5)	13.4, 24.6	0.117	154 (77.0)	46 (23.0)	17.4, 29.5	0.024	200
Yes	35 (71.4)	14 (28.6)	16.6, 43.3		30 (61.2)	19 (38.8)	25.2, 53.8		49
*Difficulty eating/drinking*									
No	113 (83.7)	22 (16.3)	10.5, 23.6	0.075	110 (81.6)	25 (18.5)	12.4, 26.1	0.003	135
Yes	85 (74.6)	29 (25.4)	17.8, 34.5		74 (64.9)	40 (35.1)	26.4, 44.6		114
*Difficulty relaxing/sleeping*									
No	155 (83.3)	31 (16.7)	11.6, 22.8	0.010	144 (77.4)	42 (22.6)	16.8, 29.3	0.03	186
Yes	43 (68.3)	20 (31.7)	20.6, 44.7		40 (63.5)	23 (36.5)	24.7, 49.6		63
*Difficulty speaking/pronouncing*									
No	151 (81.2)	35 (18.8)	13.5, 25.2	0.263	144 (77.4)	42 (22.6)	16.8, 29.3	0.03	186
Yes	47 (74.6)	16 (25.4)	15.3, 37.9		40 (63.5)	23 (36.5)	24.7, 49.6		63
*Difficulty cleaning mouth*									
No	112 (85.5)	19 (14.5)	9.0, 21.7	0.014	105 (80.2)	26 (19.8)	13.4, 27.7	0.018	131
Yes	86 (72.9)	32 (27.1)	19.4, 36.0		79 (66.9)	39 (33.1)	24.7, 42.3		118
*Difficulty smiling/laughing*									
No	158 (81.9)	35 (18.1)	13.0, 24.3	0.088	150 (77.7)	43 (22.3)	16.6, 28.8	0.011	193
Yes	40 (71.4)	16 (28.6)	17.3, 42.2		34 (60.7)	22 (39.3)	26.5, 53.3		56
*Difficulty going to school/learning in class/doing homework*									
No	152 (81.3)	35 (18.7)	13.4, 25.1	0.231	143 (76.5)	44 (23.5)	17.7, 30.3	0.108	187
Yes	46 (74.2)	16 (25.8)	15.5, 38.5		41 (66.1)	21 (33.9)	22.3, 47.0		62
*Difficulty socialising with friends*									
No	158 (81.4)	36 (18.6)	13.4, 24.6	0.157	148 (76.3)	46 (23.7)	17.9, 30.3	0.106	194
Yes	40 (72.7)	15 (27.3)	16.1, 41.0		36 (65.5)	19 (34.5)	22.2, 48.6		55
Total	198 (78.5)	51 (20.5)	15.7, 26.0		184 (73.9)	65 (26.1)	20.8, 32.0		249

Regression analysis carried out to identify determinants for medication use, controlling for race, and significant pain characteristics and social impacts did not yield any independent significant determinants, with the model accounting for 17% of the variance (Table 5). For determinants of dental visits, Indians (OR = 4.43, 95% CI 1.95–10.08) and Chinese (OR = 3.46, 95% CI 1.10–10.88) were more likely to visit a dentist for tooth pain when compared to Malays. Those who reported pain medication use were also more likely to report visiting a dentist for tooth pain (OR = 4.04, 95% CI 1.86–8.79). The model accounted for 22% of the variance (Table 6).

**Table 5:** Unadjusted and Adjusted Odds Ratios (ORs) with 95% Confidence Intervals (95% CIs) for the association between medication use for tooth pain in the past 1 month and race, pain symptoms and social impacts.

	Unadjusted OR (95% CI)	*p* value	Adjusted OR (95% CI)	*p* value
*Race*				
Malay	1		1	
Chinese	0.52 (0.26–1.04)	0.066	0.63 (0.30–1.33)	0.222
Indian	1.89 (0.76–4.67)	0.169	2.14 (0.76–6.00)	0.148
*Pain intensity*				
Slight	1		1	
Moderate	1.03 (0.52–2.06)	0.925	0.74 (0.35–1.57)	0.429
Severe	4.77 (1.78–12.77)	0.002	2.00 (0.63–6.42)	0.242
*Pain worse on chewing/eating*				
No	1		1	
Yes	2.04 (1.09–3.83)	0.026	1.50 (0.75–3.01)	0.251
*Difficulty swallowing*				
No	1		1	
Yes	1.99 (0.95–4.19)	0.069	1.89 (0.82–4.36)	0.135
*Difficulty with sleeping*				
No	1		1	
Yes	2.95 (1.52–5.71)	0.001	1.80 (0.76–4.27)	0.178
*Difficulty relaxing*				
No	1		1	
Yes	2.33 (1.21–4.48)	0.012	1.22 (0.54–2.76)	0.638
*Difficulty in cleaning mouth*				
No	1		1	
Yes	2.19 (1.16–4.13)	0.015	1.65 (0.82–3.32)	0.160
*Difficulty smiling/laughing*				
No	1		1	
Yes	1.81 (0.91–3.59)	0.091	1.12 (0.51–2.46)	0.771
Nagelkerke R Square = 0.170

**Table 6:** Unadjusted and Adjusted Odds Ratios (ORs) with 95% Confidence Intervals (95% CIs) for the association between visiting a dentist for tooth pain in the past 1 month and race, pain symptoms and social impacts.

	Unadjusted OR (95% CI)	*p* value	Adjusted OR (95% CI)	*p* value
*Race*				
Malay	1		1	
Chinese	2.26 (1.18–4.32)	0.013	6.42 (2.58–15.98)	0.001
Indian	2.91 (1.14–7.43)	0.020	8.10 (2.38–27.60)	0.001
*Pain intensity*				
Slight	1		1	
Moderate	1.37 (0.72–2.60)	0.334	0.99 (0.46–2.10)	0.884
Severe	7.28 (2.67–19.83)	0.001	2.42 (0.67–8.90)	0.199
*Pain worse on chewing/eating*				
No	1		1	
Yes	1.73 (0/97–3.10)	0.065	1.20 (0.59–2.47)	0.615
*Gums swollen*				
No	1		1	
Yes	2.30 (1.19–4.69)	0.015	1.44 (0.56–3.68)	0.452
*Difficulty swallowing*				
No	1		1	
Yes	2.55 (1.28–5.10)	0.008	1.98 (0.82–4.77)	0.130
*Difficulty sleeping*				
No	1		1	
Yes	5.64 (2.99–10.64)	0.001	10.86 (4.04–29.20)	0.001
*Difficulty playing*				
No	1		1	
Yes	2.12 (1.09–4.11)	0.026	0.77 (0.27–2.23)	0.633
*Difficulty eating/drinking*				
No	1		1	
Yes	2.38 (1.33–4.25)	0.003	0.98 (0.43–2.21)	0.957
*Difficulty relaxing*				
No	1		1	
Yes	1.97 (1.06–3.66)	0.031	0.48 (0.20–1.18)	0.487
*Difficulty speaking/pronouncing*				
No	1		1	
Yes	1.97 (1.06–2.66)	0.031	1.01 (0.42–2.47)	0.976
*Difficulty cleaning mouth*				
No	1		1	
Yes	1.99 (1.12–3.55)	0.019	1.95 (0.91–4.15)	0.085
*Difficulty socialising*				
No	1			
Yes	1.70 (0.89–3.24)	1.090		
Nagelkerke R Square = 0.346				

## Discussion

This paper reports on the role of racial identity on pain medication use and dental visits for tooth pain experienced in the past 1 month among 14–18-year-olds attending four government schools in a district on the outskirt of Kuala Lumpur, Malaysia. The key findings were that tooth pain in the past 1 month was reported by more than one in five Malay and Indian adolescents, but less than one in five Chinese adolescents. Of those experiencing tooth pain, Chinese and Indian adolescents were more likely to visit a dentist for the pain compared to Malay adolescents, and those who reported medication use were also more likely to visit a dentist compared to those who did not use medication. The relationship between race and dental visits would appear to be accentuated by impact on social functioning.

The finding that racial differences in the tooth pain experience exist in Malaysian adolescents is consistent with reports from other countries.^16–18^ These differences may reflect racial variations in the caries experience, as tooth pain as a consequence of dental caries is common.^26^ Certainly racial variations in caries experience in Malaysia have been reported,^27^ but this is not conclusive.^28^ However, higher caries prevalence does not necessarily mean higher likelihood for tooth pain, as this depends on the propensity to seek treatment for caries before symptoms develop. Thus, a lower caries prevalence in a race may result in a higher tooth pain prevalence because of symptomatic care-seeking behaviour. Other factors such as dental fear, pain coping mechanisms, access to care, and socio-economic position associated with race may be implicated. Viewed historically as a biological construct, race is now more usually considered a social–psychological and social–political construct.^29^ In this sense, it is regarded as a co-occurrence with ethnicity, and is used to understand the health consequences of variations in factors such as healthcare quality and utilization, housing, education, and nutrition. Although racial and ethnic differences in health and disease may be related to socio-economic status, culture, and environmental and genetic influences,^21^ some researchers have suggested that these differences can be independent of social economic status.^30^

Of the adolescents in the present study who reported tooth pain in the past 1 month, only a fifth used pain medication and a quarter visited a dentist, consistent with orofacial pain related care-seeking for adults.^31^ Of these, Chinese and Indians were more likely than Malays to visit a dentist for tooth pain. The literature on racial variations in dental utilisation patterns of children and adolescents for tooth pain is limited, with some data having emerged from New Zealand.^13,17^ These disparities may reflect race-related socio-economic position but may also reflect cultural differences in pain coping.^32^ Access to dental care may be a key factor and an understanding of the design of dental service provision is needed to determine if certain racial groups are disadvantaged. This is important in order to avoid dental emergences in hospital emergency departments.^33^

The findings in this study should be considered in the context of its methodological limitations. We did not collect socio-economic data on the sample, and therefore the role of race-related socio-economic status was not known. However, the sample was drawn from similar government schools in the same multi-racial geographical district, thus we considered the sample to be homogeneous.

## Conclusion

Racial variations in the tooth pain experience and care-seeking have been identified in the Malaysian context, with Indians and Malays more likely than Chinese to experience tooth pain, but Chinese and Indians more likely than Malays to seek care. This may be related to socio-economic status, but connotations of culture-related care-seeking and accessibility of dental services should not be ignored. The identification of race alone did not allow us to explore the complex multitude of inter and intra race-related factors that are associated with the tooth pain experience and related care-seeking patterns. Further research is needed to understand these factors, which may include pain coping mechanisms and perceptions of service designs in order to address the scope that tooth pain poses as a dental public health problem.
